# The Role of Pericytes in Ischemic Stroke: Fom Cellular Functions to Therapeutic Targets

**DOI:** 10.3389/fnmol.2022.866700

**Published:** 2022-04-13

**Authors:** Sheng-Yu Zhou, Zhen-Ni Guo, Dian-Hui Zhang, Yang Qu, Hang Jin

**Affiliations:** ^1^Department of Neurology, Stroke Center, The First Hospital of Jilin University, Changchun, China; ^2^Clinical Trial and Research Center for Stroke, Department of Neurology, The First Hospital of Jilin University, Changchun, China

**Keywords:** pericyte, ischemic stroke, neurovascular unit, brain-blood barrier, therapeutic targets

## Abstract

Ischemic stroke (IS) is a cerebrovascular disease causing high rates of disability and fatality. In recent years, the concept of the neurovascular unit (NVU) has been accepted by an increasing number of researchers and is expected to become a new paradigm for exploring the pathogenesis and treatment of IS. NVUs are composed of neurons, endothelial cells, pericytes, astrocytes, microglia, and the extracellular matrix. As an important part of the NVU, pericytes provide support for other cellular components and perform a variety of functions, including participating in the maintenance of the normal physiological function of the blood–brain barrier, regulating blood flow, and playing a role in inflammation, angiogenesis, and neurogenesis. Therefore, treatment strategies targeting pericyte functions, regulating pericyte epigenetics, and transplanting pericytes warrant exploration. In this review, we describe the reactions of pericytes after IS, summarize the potential therapeutic targets and strategies targeting pericytes for IS, and provide new treatment ideas for ischemic stroke.

## Introduction

**FIGURE 1:**
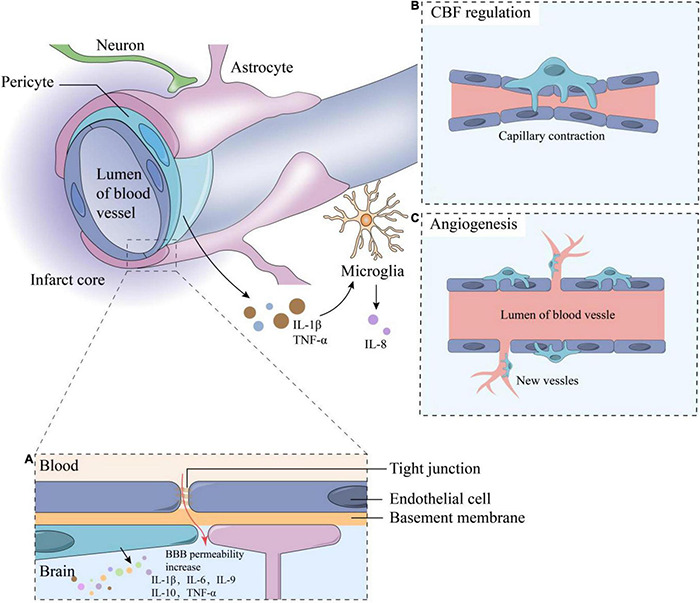
The composition of neurovascular units and functions of pericytes. The components of NVU including neurons, astrocytes, microglia, vascular endothelial cells, pericytes, basement membrane and extracellular matrix, and adjacent endothelial cells are connected by tight junctions. Some functions of pericytes including: **(A)** Inflammation response: when IS occurs, the BBB permeability increase, stimulating the inflammatory responses and cytokine secretion of pericytes such as IL-1β, IL-6, IL-9, IL-10, etc.; **(B)** CBF regulation; **(C)** Angiogenesis. TNF-α and IL-1β secreted by pericytes can stimulate microglia to secrete IL-8. CBF, cerebral blood flow; BBB, blood-brain barrier; TNF, tumor necrosis factor; IL, interleukin.

Ischemic stroke (IS) is a cerebrovascular disease with high morbidity, disability, and mortality rates and accounts for about 87 % of all strokes, it occurs when a vessel supplying blood to the brain is obstructed, which leads to the reduction of blood flow and a sudden loss of brain function, while hemorrhagic stroke is due to bleeding into the brain by the rupture of a blood vessel (Datta et al., 2020; Steliga et al., 2020). IS is accompanied by a series of molecular and cellular events, which include immune inflammatory response, glial cell activation, blood–brain barrier (BBB) destruction, and neuronal cell death. Previous treatments for IS focused on vascular recanalization and neuroprotection (Molina and Saver, 2005; Nguyen et al., 2021); however, recent studies have shown that the outcome of these treatments may be affected by the components of the neurovascular unit (NVU) (ElAli et al., 2014; Morrison and Filosa, 2019; Cao et al., 2021; Le Roy et al., 2021; Wang L. et al., 2021). Therefore, targeting the NVU is being approached as a new treatment strategy for IS. NVU forms the fundamental structural and functional unit of the central nervous system (CNS). As shown in Figure 1, NVU is composed of neurons, basal lamina, endothelial cells (ECs), pericapillary microglia, astrocytes, and pericytes. Among these components, pericytes, which are also known as parietal cells, are widely distributed in the microvascular system. They are embedded in the basal membrane of ECs and are one of the components of the microvascular wall (Abbott et al., 2010; Obermeier et al., 2013; Daneman and Prat, 2015). Although there are peripheral pericytes, the CNS pericytes is mostly involved in the physiological process and in the pathological process after IS.

Central nervous system pericytes are derived from different embryonic sources during development. According to a series of bird chimerism studies, quail neuroectoderm or mesoderm was transplanted into developing chicken embryos. Transplanted neuroectoderm was found to give rise to pericytes in the forebrain, while transplanted mesoderm gave rise to pericytes in the midbrain, brain stem, spinal cord and peripheral organs (Etchevers et al., 2001). This result suggests that pericytes in the forebrain are derived from neural crest cells, while those in the midbrain, brainstem, and spinal cord are derived from mesenchymal stem cells (MSCs) (Winkler et al., 2011).

Pericytes in the CNS are classified into three types: arterial, capillary, and venule (Attwell et al., 2016). These three pericyte subtypes play critical roles in normal brain function, brain injury, and functional recovery after stroke. Arterial pericytes are located at the end of arterioles in the capillary bed and exhibit strong contractility due to the expression of high levels of α-smooth muscle actin (α-SMA), which play a key role in regulating cerebral blood flow. Capillary pericytes are important for maintaining the integrity of the BBB, which are located between the capillary beds and express less α-SMA than arterial pericytes. Pericytes of venules are located at the ends of capillaries and regulate the infiltration of peripheral immune cells into the brain parenchyma. Pericytes can provide support for other NVU components including astrocytes, endothelial cells, microglia and neuron, and maintain the normal physiological function of the BBB. They are also involved in the regulation of cerebral blood flow, repairment of BBB, immune inflammatory response, angiogenesis, and neurogenesis. Epigenetic regulation of pericytes is expected to be a new therapeutic target for IS. Since pericytes are abundant in the microvasculature and are involved in a variety of vascular functions, alterations in pericyte coverage or pericyte dysfunction may contribute to vascular dysfunction. Microvascular pericytes are lost during ischemia/reperfusion after IS, in which oxidative stress plays an important role and ROS-induced cell damage may be the main cause of pericyte apoptosis, and pericytes are more susceptible to oxidative stress than ECs (Hill et al., 2014; Ferland-McCollough et al., 2017). Since oxidative stress plays an important role in this process, free radical scavengers such as edaravone can be considered as potential therapeutic strategies for IS (Deguchi et al., 2014). However, there are few studies on the role of pericytes in hemorrhagic stroke at present, so we focus on the role of pericytes in IS. This review summarizes the function of pericytes in IS and explores potential therapeutic targets and treatment strategies based on pericytes.

## The Role of Pericytes in Ischemic Stroke

Pericytes participate in multiple pathophysiological processes in the NVU during the occurrence of ID. As an important component of the NVU, pericytes provide support for other NVU constituent cells, control the blood flow of the capillaries of the CNS, and maintain the integrity of the BBB. Hence, they help in maintaining the normal function of NVU. Pericytes are also involved in ischemia-induced angiogenesis and neurogenesis, and notably, in the immune response in the brain after IS. Mechanistically, many of these functions are achieved by epigenetic regulation.

### Pericytes in BBB Repair

The BBB is composed of ECs, basal lamina, astrocytes, and pericytes. it maintains homeostasis of the brain microenvironment by controlling the entry and exit of certain substances in the brain (Bergers and Song, 2005; Keaney and Campbell, 2015). Hence, BBB destruction is an important pathological change that occurs in IS (Keaney and Campbell, 2015). In the development of cerebral microcirculation, pericytes contribute to the formation and maintenance of BBB. A low count of pericytes greatly increases the permeability of BBB, further confirming that pericytes are key to maintaining its integrity (Bell et al., 2010). Winkler et al. demonstrated that platelet-derived growth factor-B/platelet-derived growth factor receptor-B (PDGF-B/PDGFR-B) can mediate pericyte recruitment to the vessel walls in the embryonic brain and regulate the development of cerebral microcirculation and the BBB. They used mouse models lacking PDGF-B and PDGFR-B to investigate whether the loss of pericytes can influence brain capillary density, and found that loss of pericytes can disrupt cerebral microcirculation and BBB integrity, which occur in neurodegenerative and neurovascular disorders (Winkler et al., 2010). In addition, pericytes synergize with other components of the BBB such as ECs, basal lamina, and astrocytes (Gundersen et al., 2014). In mice with defective pericyte generation, structural abnormalities in tight junctions were observed using electron microscopy. Further, the permeability of BBB also increased (Daneman et al., 2010). Trans-endothelial electrical resistance (TEER) is a widely accepted quantitative technique for measuring the integrity of tight junction dynamics in endothelial monolayer cell culture models. It is an indicator that can provide a measure of pore access through ECs and the integrity of cell-to-cell junctions. TEER can serve as an excellent tool to elucidate endothelial barrier functions, such as responses to cytokines, pathological stimuli, and potential therapeutic agents (Ramnath and Satchell, 2020). It is a strong indicator often used to evaluate BBB integrity, and an increase in TEER usually indicates a decrease in BBB permeability (Srinivasan et al., 2015; Vatine et al., 2019). Glial cell line-derived neurotrophic factor (GDNF) facilitates neuronal or axonal regeneration, and increases the expression of claudin-5 and the TEER value of ECs. Shimizu et al. demonstrated that GDNF secreted from pericytes of the brain and peripheral nerves upregulated the expression of claudin-5, the main protein that constitutes the tight junction of ECs, which increased the TEER value of microvascular ECs (Shimizu et al., 2012). This result confirmed that pericytes are essential for maintaining BBB permeability, as claudin-5 is now considered to be one of the most important components involved in maintaining BBB function (Nitta et al., 2003; Dithmer et al., 2017). Hence, targeting GDNF secreted by pericytes may be a new therapeutic strategy to alter the function of the BBB and promote nerve regeneration.

Another study illustrated that in a gel matrix, pericytes and astrocytes migrate together and adhere to capillary-like structures to form glial vascular complex-like structures. Compared with astrocytes alone, pericytes in combination with astrocytes inhibited the degradation of the capillary-like structures to a greater extent (Itoh et al., 2011). Furthermore, various studies have shown that pericytes are involved in the formation and degradation of extracellular matrix (ECM) in the basal layer. Pericytes produce degrading proteases, such as matrix metalloproteinase (MMP)-2 and MMP-9, which can promote the degradation of ECM in the early stage of angiogenesis. This leads to the detachment of pericytes from basal lamina and their migration to newly formed vascular, helping repair the damaged BBB after IS (Zozulya et al., 2008; Takata et al., 2011; Jullienne et al., 2016). During the vascular stabilization process, pericytes express inhibitors of MMP such as the metalloproteinase-3 tissue inhibitor to inhibit the degradation of ECM and promote vascular maturation and BBB repair (Dalkara et al., 2011; Winkler et al., 2011). In contrast, the structural and functional changes in pericytes, such as the occurrence of oxidative stress in the acute stage of brain ischemia, can also lead to the destruction of the BBB and accumulation of neurotoxic substances. Oxidative stress also activates pericytes to secrete MMP-9 and other factors. Activated MMP-9 induces pericyte damage, shedding, and migration. These factors further aggravate adhesion damage (Nishimura et al., 2016). Thus, in the early stage of ischemia, pericytes and the factors secreted by them may lead to disruption of the BBB. This evidence illustrated that pericytes undergo a two-way regulation. Therefore, inhibiting the secretion of MMP-9 by pericytes in the acute stage of IS and promoting the expression of MMP inhibitors secreted by pericytes during vascular stabilization process can be considered to be possible therapeutic strategies of IS.

### Pericytes in Cerebral Blood Flow Regulation

Pathological changes in smooth muscle cells after IS can interrupt cerebral blood flow (CBF). Studies have shown that pericytes have potential contractility and can regulate the blood supply to terminal vessels with damaged smooth muscle cells (Liu et al., 2021; Mariana et al., 2021). Pericytes express the contractile protein α-SMA, which dilates and contracts blood vessels, participates in neurovascular coupling, and regulates CBF (Cheng et al., 2018). Therefore, α-SMA is a commonly used pericyte marker, and higher expression of α-SMA indicates stronger contractility (Kelly-Goss et al., 2014). Some studies have indicated that transforming growth factor-β (TGFβ) increases the expression of α-SMA (Shih et al., 2003), whereas fibroblast growth factor-2 (FGF-2) has the opposite effect (Papetti et al., 2003). Regulating the contractility of pericytes and microvascular blood flow by regulating the expression of α-SMA could be considered as a potential strategy for IS treatment. Notably, the hyperconstriction of blood vessels is pathological and is facilitated by smooth muscle and pericytes. Pericyte contraction is more pronounced during reperfusion after IS. Reperfusion may cause more damage to the injured tissue than ischemia itself. Among them, oxidative stress is an effective contraction inducer of pericytes and is a particularly prominent damage mediator in ischemia-reperfusion injury (O’Farrell et al., 2017). Yemisci et al. (2009) reported contractility changes in pericytes during reperfusion in IS *in vivo*. Pericyte contraction occurs 1 h after reperfusion in a stroke middle cerebral artery occlusion model. It has been shown that pericyte contraction is induced by oxidative and nitrifying free radicals before reperfusion relieves pericyte contraction (Yemisci et al., 2009). This study demonstrates the importance of pericyte contractility in stroke pathology and suggests that persistent contraction of pericytes may be responsible for the poor outcomes of thrombolytic therapy in stroke.

Pericytes also contribute to the cerebral no-reflow phenomenon in which microcirculation cannot be fully restored after the occluded blood vessel is reopened (Kloner et al., 2018). Electron microscopic examination of the cerebral microvascular system in the ischemic area showed capillary damage manifested as swollen ECs and astrocytic ends, intravascular platelets and fibrin, and white blood cell blockage (McCabe et al., 2018). These changes compress capillaries, leading to the persistence of microcirculation disorders (Paljärvi et al., 1983; Taskiran-Sag et al., 2018). Pericytes remained contracted during ischemia even after the main blood supply artery was successfully reopened, which may be the main reason for the non-reflux phenomenon (Yemisci et al., 2009; Dalkara and Alarcon-Martinez, 2015). Hall et al. (2014) suggested that cerebral ischemia-induced pericyte contraction and the subsequent death of pericytes. The contraction of pericytes is regulated by intracellular Ca^2+^ concentration. Upon cerebral ischemia, a large Ca^2+^ influx triggers calcium overload and contraction of pericytes, causing cerebral microvasculature contraction. The subsequent death of pericytes leaves the microvasculature in a continuous state of contraction, which causes microcirculation failure. During reperfusion, pericytes produce a large amount of reactive oxygen species (ROS) and calcium overload is aggravated, whereas suppressing the reperfusion-induced increase in ROS can promote microcirculation reperfusion (Taskiran-Sag et al., 2018). Therefore, preventing the contraction and death of pericytes as soon as possible after IS may help in the recovery of microvascular CBF, promote microcirculation reperfusion, and improve the outcome of IS.

### Pericytes in the Inflammation Response

Pericytes have various properties of immune regulatory cells, including leukocyte recruitment, reactive secretion of inflammatory factors, antigen presentation, and phagocytosis. They also participate in many aspects of CNS inflammatory responses (Lange et al., 2013; Pieper et al., 2013, 2014; ElAli et al., 2014). In the inflammatory response after IS, pericytes play both pro-inflammatory and immunoregulatory roles. In pericyte-deficient mice (*PDGFRβ^–/–^*), intercellular adhesion molecule-1 (ICAM-1) is significantly upregulated, and leukocytes, which are normally absent from the CNS, are present. This indicates that pericytes can protect the brain from the invasion of leukocytes. Pericytes also show phagocytic and migratory characteristics after IS (Rustenhoven et al., 2018). Studies have shown that brain microvascular pericytes express damage-associated molecular patterns and toll-like receptor 4 (Guijarro-Muñoz et al., 2014), triggering an immune inflammatory response. In addition, several *in vitro* studies have shown that various inflammatory conditions can induce pericytes to express multiple cytokines, including interleukin (IL)-9, IL-10, and tumor necrosis factor-α (TNF-α) (Lange et al., 2013; Pieper et al., 2013, 2014; Liu et al., 2016). In addition, a variety of inflammatory conditions can also induce pericytes to express pro-inflammatory factors as shown by several *in vitro* studies. For example, TNF-α and IL-1β stimulate microglia to secrete the chemokine IL-8 (Pieper et al., 2013); consequently, TNF-α and TGFβ1 enhance the expression of IL-6 in human pericytes, which can disrupt BBB functioning (Rustenhoven et al., 2016). Pericytes can also upregulate the expression of ICAM-1 and vascular cell adhesion molecule-1 in response to interferon-γ (Balabanov et al., 1999; Lyck and Enzmann, 2015). Therefore, pericytes may be considered as effectors and amplifiers of the inflammatory process in the brain during IS.

Pericytes can transport substances through phagocytosis, pinocytosis and endocytosis and participate in the first line of immune defense in the brain after IS (Thomas, 1999; Girolamo et al., 2021). Pericytes may transform into other cell types to participate in inflammation and immune processes during the occurrence of IS. Pericytes can differentiate into various cell types such as microglia-like cells with phagocytic capacity, which are involved in the immune inflammatory response in IS (Sakuma et al., 2016). This evidence indicates that pericyte loss may be the cause of aggravation of leukocyte infiltration during IS.

### Pericytes in Angiogenesis

**FIGURE 2:**
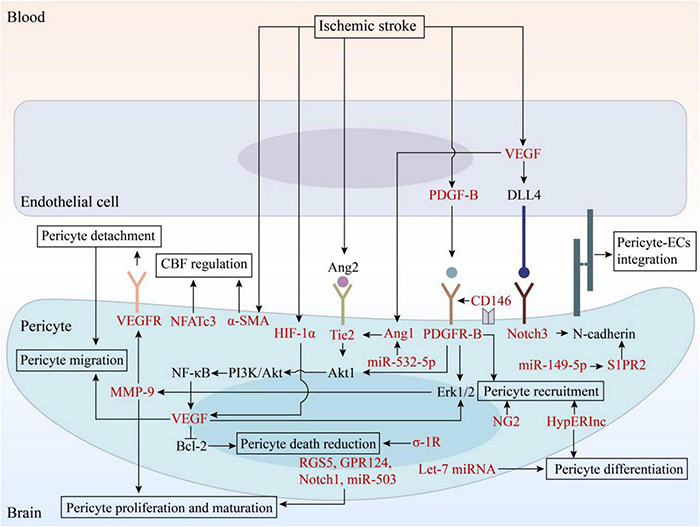
The signaling pathways involved in angiogenesis and potential therapeutic targets. EC, endothelial cell; VEGF, vascular endothelial growth factor; VEGFR, vascular endothelial growth factor receptor; PDGF, platelet-derived growth factor; PDGFR, platelet-derived growth factor receptor; Ang, angiopoietin; DLL4, Delta-like ligand 4; HIF, hypoxia-inducible factor; MMP, matrix metalloproteinase; NF-κB, nuclear factor kappa-B; Bcl-2, B cell lymphoma-2; PI3K, phosphatidylinositol 3-kinase; RGS5, regulator of G-protein signaling 5; GPR124, G protein-coupled receptor 124; σ-1R, sigma-1 receptor; NG2, neuron-glial 2; α-SMA, α-smooth muscle actin; NFATc3, nuclear factor of activated T-cells, cytoplasmic 3; HypERlnc, hypoxia-induced endoplasmic reticulum stress regulating lncRNA; S1PR2, sphingosine-1-phosphate receptor 2.

In IS, the microcirculation of the ischemic brain tissue cannot be fully perfused after recanalization. This phenomenon, termed reperfusion no-reflow, is harmful to the recovery of ischemic tissues. However, angiogenesis promotes the accumulation of new neuronal cells in the peri-infarct area and contributes to the repair of brain injury after IS, which is significant for tissue regeneration and provides new entry for blood reflow to the hypoperfusion area (Di et al., 2021). The remodeling of microvascular system after IS includes four stages: proliferation of vascular component cells; recruitment of pericytes; formation, migration, and stabilization of vascular budding; and maturation of blood vessels (Stapor et al., 2014; Yang and Torbey, 2020). Pericytes regulate all these stages through a dynamic balance between pro-angiogenic and anti-angiogenic factors. In recent years, pericytes have received increasing attention as potential targets for pro-angiogenesis therapy after stroke. During angiogenesis, bidirectional EC-pericyte signaling is essential (Winkler et al., 2011; Hall et al., 2014; Iadecola, 2017). First, after exposure to pro-angiogenic stimuli, pericytes upregulate Erk1/2-MMP axis to degrade basal membrane components, leading to the detachment of pericytes and subsequent release of ECs and increased vascular permeability (Carmeliet and Collen, 2000; Potente et al., 2011; Xiao et al., 2020). This promotes the extravasation of plasma proteins and provides a stent for the production of vascular sprouts (Carmeliet and Collen, 2000; Zhu et al., 2021). Second, pericytes secrete vascular endothelial growth factor (VEGF)-A, which stimulates ECs to transform into tip cells. Further, stem cells proliferate to support the elongation of vascular sprouts and form blood vessels. In this process, pericytes attach to the ECs, regulate the formation of the extracellular matrix and tight junctions between endothelial cells, and maintain the integrity of immature blood vessels (Franco et al., 2011). Pericytes interact with ECs through a variety of signaling pathways to regulate angiogenesis, including the PDGF-B/PDGFR-B, angiopoietin (Ang)/Tie2, VEGF/VEGFR, Notch1, and Notch 3 signaling pathways (Berger et al., 2005; Nakamura et al., 2016; Leligdowicz et al., 2018; Özen et al., 2018; Shibahara et al., 2020a; Gong et al., 2021; Nguyen et al., 2021), and these pathways are summarized in Figure 2. Finally, the tip cells anastomose with adjacent vascular buds to form a microvascular ring. After these processes, pericytes inhibit the proliferation of ECs by downregulating the expression of VEGF and establishing a resting state in ECs to stop angiogenesis (Jain, 2003; Huang, 2020). Therefore, further research for the treatment of IS can help in developing new therapies that promote angiogenesis.

### Pericytes in Neurogenesis

Neurogenesis can be observed in the brain during IS (Rüber and Schlaug, 2018; Hatakeyama et al., 2020; Yang and Torbey, 2020; Zhan et al., 2020). A study reported that neural stem cells developed in the injured areas of a mouse model of transient brain ischemia/reperfusion injury. Surprisingly, some PDGFR-B+ pericytes can be a potential source of perivascular neural stem cells, which have the potential to generate neurons and repair the NVU in patients with IS. Bone marrow-derived cells that have a phenotype of pericytes and express angiogenic factors such as VEGF, are recruited to the brain capillaries. Accumulating evidence shows that angiogenesis is closely connected with neurogenesis. Further, newly formed neurons are always located near the remodeled vessels, probably because the vascular cells recruit and form the niche of neural stem cells. Some angiogenic factors, such as VEGF and PDGF, also play a vital role in neurogenesis after stroke (Bai et al., 2015; Eilken et al., 2017; Caporarello et al., 2019; Nguyen et al., 2021). VEGF can protect pericytes and NVUs from IS by enhancing the MEK1/2/ERK1/2 and PI3K/AKT signaling pathways, which triggers a part of the feedforward loop of IS. Wang et al. showed that after IS, the PDGF/PDGFR-B signaling pathway is essential for the recruitment of neuroblasts from the subventricular zone to the infarct area in a mouse model (Wang et al., 2017). They downregulated the PDGFR signaling pathway with crenolanib, a nonselective PDGFR inhibitor, and found that crenolanib increased pericyte apoptosis, decreased pericyte and vascular coverage, increased neuroblast apoptosis, and inhibited neuroblast migration in the subventricular zone. These results indicated that the PDGF/PDGFR-B signaling pathway can promote the migration and survival of neuroblasts after IS and affect stroke-induced neurogenesis. Furthermore, it has also been reported that pericytes can stabilize quiescent microvasculature, enrich local neuronal microcircuits, and promote neurogenesis (Farahani et al., 2019). Pericytes with neuro-angiogenic potential can be considered as potential targets for repairing the damaged CNS after IS.

## Potential Treatment Strategies and Therapeutic Targets of Pericytes in Is

Since pericytes mediate important pathological processes in IS, the development of therapeutic strategies that target pericytes is presently a focus of research for treating IS. Treatments for IS that target pericytes are summarized in Table 1. The drugs used for targeting pericytes in IS are listed in Table 2. Potential therapeutic strategies for IS include promoting the proliferation and maturation of pericytes, reducing death and increasing survival of pericytes, and regulating the recruitment of pericytes after IS, among others.

**TABLE 1:** Potential targets of treating IS that target pericytes.

Strategies	Targets	Functions	References
Promote the proliferation and maturation of pericytes	Matrix metalloproteinase (MMP)	Enables pericytes to detach from basal lamina and migrate to newly formed microvasculature and balances the degradation and maturation of vascular after IS	Zozulya et al., 2008; Takata et al., 2011; Shimizu et al., 2012; Jullienne et al., 2016
	Regulator of G-protein signaling 5 (RGS5)	Hinders pericytes proliferation and contributes to pericyte maturation	Berger et al., 2005
	angiopoietin (Ang)/Tie2	Participates in proliferation of pericytes and ECs and the maturation of new blood vessels	Chakroborty et al., 2011; Leligdowicz et al., 2018
	G protein-coupled receptor 124 (GPR124)	Promotes the filopodia formation and cell migration of pericytes	Chen et al., 2019
	Notch 1	Increase the coverage of pericytes and maintains vascular stability	Wang et al., 2014
Reduce the death and increase the survival of pericytes	vascular endothelial growth factor (VEGF)	promote angiogenesis and stabilize the cerebral microvascular system	Benjamin et al., 1998; Darden et al., 2019
	platelet-derived growth factor β/platelet-derived growth factor receptor β (PDGFβ/PDGFRβ)	induce cell growth and anti-apoptotic responses	Makihara et al., 2015; Trost et al., 2016; Shibahara et al., 2020a,b
	Hypoxia-inducible factor-1 (HIF-1)	The absence of HIF-1 can maintain the integrity of the BBB by reducing the death of pericytes	Tsao et al., 2021
	The sigma-1 receptor (σ-1R)	Reduces cell apoptosis significantly by inhibiting autophagy, subsequently increases pericyte survival and reduced BBB damage	Zhang et al., 2020
Regulate the recruitment of pericytes	Neuron-glial 2 (NG2)	Plays an key role in pericytes recruitment and the interaction with ECs during microvascular development	Chekenya et al., 2002; Ozerdem et al., 2002; Ozerdem and Stallcup, 2004; You et al., 2012
	CD146	Functions as a coreceptor of PDGFR-β, mediates pericyte recruitment to cerebrovascular ECs and help angiogenesis	Chen et al., 2017
Control the regulation of CBF	α-smooth muscle actin (α-SMA)	dilates and contract blood vessels, participates in neurovascular coupling, and regulating CBF	Thanabalasundaram et al., 2011
	Nuclear factor of activated T-cells, cytoplasmic 3 (NFATc3)	Regulates vascular cell contractility and CBF	Filosa et al., 2007; Serrano-Pérez et al., 2011
Regulate epigenetics	miR-149-5p	Increases the expression of N-cadherin, reduces pericyte migration and the permeability of BBB	Wan et al., 2018
	miR-532-5p	Promotes pericyte coverage, Ang-1 expression *in vivo*, and vascular maturation	Slater et al., 2018
	miR-503	The blockage of miR-503 can help increase the coverage of capillary vessels and reducing the permeability of BBB	Caporali et al., 2015
	Let-7 miRNA	involved in pericyte differentiation in response to hypoxia	Esen et al., 2016
	hypoxia-induced endoplasmic reticulum stress regulating lncRNA (HypERlnc)	Regulates the differentiation, proliferation and recruitment of pericytes to endothelial cells	Bischoff et al., 2017

**TABLE 2:** Drugs for IS that target pericytes.

Drugs	Type of trial	Development phase	Outcomes	References
Atorvastatin	Preclinical trials	Preclinical	Promotes the maturation of new blood vessels by activate the Pl3k-Akt pathway *in vitro* study	Urbich et al., 2002
	Clinical trials	Phase II and IV	80 mg of atorvastatin per day can reduce the overall incidence of stroke and cardiovascular events in patients with recent stroke or TIA and unknown coronary heart disease; atorvastatin withdrawal can be associated with the increased risk of death or dependency at 90 day	Amarenco et al., 2006; Amarenco et al., 2007
Cilostazol	Preclinical trials	Preclinical	Reduces the expression and activity of MMP-9 and increases the expression of VEGFR2, promoting angiogenesis and protecting NVU in rat models	Omote et al., 2014
	Clinical trials	Phase IV	Although cilostazol does not reduce the risk of hemorrhagic stroke, it is not inferior to aspirin in preventing cardiovascular events in patients with ischemic stroke at high risk of cerebral hemorrhage	Kim et al., 2018
Edaravone	Preclinical trials	Preclinical	Functions as a free radical scavenger, inhibits the production of MMP-9, recovers the number of PDGFRβ-positive pericytes, protects the integrity of the NVU and reduces the damage after IS in rat models	Deguchi et al., 2014
Perlecan	Preclinical trials	Preclinical	Regulates pericyte recruitment through the cooperative functioning of PDGFRβ, support BBB maintenance and repair after IS in mice models	Nakamura et al., 2019

### Promoting the Proliferation and Maturation of Pericytes

Several studies show that capillary PCs are rapidly lost after cerebral ischemia in both experimental and human stroke (Carmeliet and Jain, 2011; Fernández-Klett et al., 2013). As previously described, a low count of pericytes greatly increases the permeability of BBB (Bell et al., 2010), which indicates that the number of pericytes is a factor in determining BBB permeability. A study shows that after IS, a ubiquitous healing mechanism, scar formation, repairs damaged tissue by producing ECM. Coinciding with this loss was a massive proliferation of PDGFR-B+ cells, originating from the neurovascular unit and depositing ECM in the mouse brain after IS. And this fibrotic response is distinct from astrocyte scarring, which, despite some regenerative function, has a potentially deleterious function in the brain (Fernández-Klett et al., 2013). In another study of Dias et al., they report that the primary source of scar-forming fibroblasts is pericytes in a mouse model of fibrotic scar tissue formation after IS. Although it is unclear whether scar-forming stromal cells have the same origin throughout the CNS and across different types of lesions, pericyte can also be considered as a therapeutic target to improve recovery after CNS injury and need further researches (Dias et al., 2021). Therefore, promoting the proliferation and maturation of pericytes can be regarded as an potential strategies of IS.

#### Matrix Metalloproteinase

Matrix metalloproteinase is a protein that enables pericytes to detach from the basal lamina and migrate to the newly formed microvasculature. This helps in establishing a balance between degradation and maturation of the vasculature after IS. A study reported that the antiplatelet drug cilostazol reduced the expression and activity of MMP-9, increase the expression of VEGFR2, promote the detachment of pericyte and subsequent angiogenesis, protect NVUs by boosting the proliferation of pericytes, and ultimately help repair the BBB after IS in rat models (Omote et al., 2014). A multicenter, randomized controlled clinical trial for Asian patients with IS, who were at high risk of cerebral hemorrhage, found that although cilostazol did not reduce the risk of hemorrhagic stroke, it was not inferior to aspirin in preventing cardiovascular events (Kim et al., 2018). Edaravone, a free radical scavenger, can also inhibit the production of MMP-9, recover the number of PDGFRβ-positive pericytes, protect the integrity of the NVU, and reduce damage after IS in rat models (Deguchi et al., 2014). Moreover, as free radical scavenger, edaravone might suppress oxidative–nitrative stress, which prevents pericyte contraction and reduces infarct volume (Zhang et al., 2004; Deguchi et al., 2014). A study investigated the relationship between changes of pericytes in NVU and the effects of tissue plasminogen activator (tPA) and edaravone. The study demonstrated that exogenous tPA damaged pericytes, led to astrocyte detachment, reduced the secretion of glial cell line-derived neurotrophic factors, and disrupted NVU integrity, while edaravone treatment greatly ameliorated this injury after IS in rat model (Deguchi et al., 2014). These evidence indicated that MMP is a potential therapeutic target for IS.

#### Regulator of G-Protein Signaling 5

The breakdown of the BBB can exacerbate brain damage after IS. Detachment of pericytes leads to BBB destruction and neurovascular dysfunction; however, the regulation of this process in IS remains unclear. Downregulating the expression of regulator of G-protein signaling 5 (RGS5), an angiogenesis marker of pericytes, hindered pericyte proliferation and contributed to pericyte maturation (Berger et al., 2005). Another recent study in *RGS5* knockout mice demonstrated that the loss of RGS5 increased the number of pericytes and their endothelial coverage. This was associated with higher capillary density and length, and significantly reduced BBB damage after IS (Özen et al., 2018). Therefore, RGS5 has been identified as a potential target for neurovascular protection.

#### Ang/Tie

The Ang/Tie system regulates angiogenesis in both the directions. The Ang/Tie family contains three ligands (Ang1, Ang2, and Ang4) and two receptors (Tie1 and Tie2). Of these, the Ang1 and Tie2 receptors participate in pericyte recruitment and capillary maturation (Chakroborty et al., 2011; Leligdowicz et al., 2018). The phosphorylation of Tie2 that is driven by Ang1, leads to the activation of downstream pathways that mediate survival, proliferation, migration, and anti-inflammatory signals (Goel et al., 2011; Qin et al., 2013). In pericytes, both Ang 1 and Tie 2 expression increased with VEGF elevation after hypoxia, and stimulates the level of Akt1, which acts on VEGF in turn (Gurnik et al., 2016). Another ligand, Ang2, upregulates the formation of new blood vessels. As an antagonist of Ang1/Tie2 signaling, Ang2 inhibits Tie2 signaling in the presence of Ang1 and weakly stimulates Tie2 in the absence of Ang1 (Wakui et al., 2006; Teichert et al., 2017). The balance between Ang1 and Ang2 is important for the proliferation of pericytes and ECs, and the maturation of new blood vessels. Thus, adjusting the balance of angiogenesis at different stages can promote beneficial processes; hence, targeting Ang2 can serves as a potential treatment strategy for IS.

#### Notch1 and Notch3

The Notch proteins namely, Notch1 and Notch3, can regulate angiogenesis. Notch3 is expressed in zebrafish pericytes, and its mutation led to a reduction in pericyte count in a zebrafish model (Wang et al., 2014). A study reported that by blocking Notch signaling in pericytes, pericytes were lost and their coverage was significantly reduced, leading to vasodilation. This indicated that Notch signaling plays an important role in maintaining vascular stability (Kofler et al., 2015). When IS occurs, VEGF expression increases in ECs and interacts with the Notch ligand, Delta-like ligand 4 (DLL4). DLL4 attaches to the pericyte surface receptor Notch3, triggering Notch signaling, leading to the upregulation of N-cadherin, a protein that decreases BBB permeability, and maintaining pericyte-ECs integration (Schulz et al., 2015). Notch1 is considered an important regulator of angiogenesis, but its role in cerebral ischemia-induced angiogenesis remains unclear. A study illustrated that the knockout of Notch1 can eliminate ischemia-induced angiogenesis in the peripheral area of cerebral infarction in a mice model (Ren et al., 2018). Since Notch proteins can facilitate angiogenesis after IS, they can also be regarded as potential therapeutic targets for IS.

#### G Protein-Coupled Receptor 124

The long-term occlusion of multiple capillaries can cause capillary damage. It has been reported that the maintenance of CNS angiogenesis and BBB integrity is essential, and endothelial G protein-coupled receptor 124 (GPR124) is required for normal forebrain angiogenesis and BBB function in mouse embryos (Kuhnert et al., 2010). According to a study by Chen et al. (2019), the expression of GPR124 in pericytes increased after microsphere embolization, and partial deletion of the GPR124 domain reduced filopodia formation and cell migration of pericytes. These observations provided evidence for the role of GPR124 in the migration of pericytes after IS. Another study showed that a conditional knockout of GPR124 in the endothelia of adult mice did not affect homeostatic BBB integrity, but resulted in BBB disruption and microvascular hemorrhage in mouse models of both IS and glioblastoma (Chang et al., 2017). These findings illustrated that GPR124 can be regarded as a potential therapeutic target for human CNS diseases characterized by BBB destruction such as IS.

### Reducing Death and Increasing Survival of Pericytes

#### Hypoxia-Inducible Factor-1 (HIF-1)

Hypoxia-inducible factor-1 (HIF-1) is the main regulator of the injury response after IS and manifests different effects in various cells. While HIF-1 reportedly has a neuroprotective effect, it also induces vascular permeability. HIF-1 loss of function in pericytes reduced ischemic damage and barrier permeability during three days of reperfusion (Tsao et al., 2021). In the absence of HIF-1, the integrity of BBB was maintained because of the reduction of pericyte death. This maintained vascular coverage and connexin organization, and inhibited vascular remodeling. A significant improvement in sensorimotor function was observed in HIF-1 loss of function mice (Tsao et al., 2021). HIF-1α also increases the level of VEGF after IS, promoting pericyte proliferation and migration and angiogenesis. Therefore, enhancing BBB integrity by inhibiting HIF-1 activation in pericytes and/or increasing pericyte survival may be a favorable option for promoting recovery after IS associated with brain injury.

#### Sigma-1 Receptor (σ-1R)

The σ-1R signaling pathway is considered to have good neuroprotective potential in the treatment of stroke. A recent study showed that sigma-1 receptor (σ-1R) agonists can reduce cell apoptosis significantly by inhibiting autophagy. Subsequently pericyte survival increased in σ-1R knockout and wild-type mice, which reduced BBB damage (Zhang et al., 2020). This result suggested that novel σ-1R agonists may be regarded as potential therapeutic agents for treating stroke.

#### VEGF

The VEGF-A/VEGFR-2 signaling pathway plays an important role in promoting angiogenesis and neurovascular remodeling after ischemia. VEGF is expressed in the infarct area and the expression of VEGF-A and its receptor, VEGFR-2, is upregulated after IS (Ramli et al., 2018). Several studies have illustrated that low levels of VEGF-A can promote angiogenesis, but overexpression of VEGF-A limited pericyte migration (Benjamin et al., 1998; Darden et al., 2019). VEGF-B, another member of the VEGF family, is also involved in angiogenesis after IS by activating the VEGFR-1 signaling pathway (Yonekura et al., 1999; Eilken et al., 2017). The BBB impairment after IS is associated with pericyte-derived VEGF. The up-regulated Akt1 initiates the phosphatidylinositol 3-kinase (PI3K)/Akt signaling pathway, leading to nuclear factor kappa-B activation to mediate VEGF expression, promote angiogenesis, and repair the BBB (Bai et al., 2015). A recent study showed that VEGF-B stimulated the formation of a stable cerebral microvascular system in the damaged area by promoting the interaction between ECs and pericytes and reducing neuronal damage and inflammation (Jean LeBlanc et al., 2018). This study also showed that VEGF-B induced the expression of anti-apoptotic protein B-cell lymphoma 2 and the main protein involved in energy homeostasis, AMP-activated protein kinase-α, thereby promoting the survival of pericytes rather than ECs. These results show that VEGF can promote angiogenesis and stabilize the cerebral microvascular system, which can be regarded as a potential treatment for IS. 3-Hydroxy-3-methylglutaryl-CoA reductase inhibitors, also known as statins, are effective cholesterol biosynthesis inhibitors used to treat hypercholesterolemia and prevent recurrent strokes (Schachter, 2005; Christophe et al., 2020). It has a pro-angiogenic effect at low doses and anti-angiogenesis and pro-apoptotic effects at high doses for IS treatment (Font et al., 2010; Garjani et al., 2012). Some studies have shown that the low-dose-dependent pro-angiogenesis effect of atorvastatin is related to the activation of the PI3K/AKT pathway in ECs, which mediates VEGF-induced maturation of pericytes (Urbich et al., 2002; Bai et al., 2015; Wang H. J. et al., 2021; Zhang et al., 2021). A double-blind randomized controlled clinical trial showed that despite a slight increase in the incidence of hemorrhagic stroke, 80 mg of atorvastatin per day can reduce the overall incidence of stroke and cardiovascular events in patients with recent stroke or transient ischemic attack and undetected coronary heart disease (Amarenco et al., 2006). Another controlled randomized study also indicated that statin withdrawal could be associated with an increased risk of death or dependency at day 90 of administration (Amarenco et al., 2007). This evidence demonstrates that atorvastatin can be regarded as an efficient drug that targets VEGF for the treatment of IS.

#### PDGF/PDGFR

Platelet-Derived Growth Factor-B plays a significant role in neuroprotection and vascular stability. PDGF transmits signals through tyrosine kinase receptors to promote pericyte recruitment to ECs. PDGF interacts with its receptor PDGFRβ, one of the most widely used pericyte markers, and contributes to angiogenesis (Makihara et al., 2015; Trost et al., 2016; Shibahara et al., 2020a,b). One study proved that PDGFRβ expression was induced and upregulated specifically in the pericytes in peri-infarct areas in a rat middle cerebral artery occlusion model (Arimura et al., 2012). This study further illustrated that PDGFβ could induce cell growth and anti-apoptotic responses, and significantly increase the expression of nerve growth factor and neurotrophin-3 through activation of Akt1 in pericytes, thereby promoting the process of angiogenesis. According to a study by Nakamura et al. (2019), perlecan, a major heparan sulfate proteoglycan of basement membranes, could regulate pericyte recruitment through a cooperative functioning with PDGFRβ, and support BBB maintenance and repair after IS.

### CBF Regulation

The recanalization of blood vessels and restoration of CBF as early as possible are currently recognized as effective methods for the treatment of acute IS. Therefore, regulating CBF by targeting pericytes is a potential therapeutic strategy for the treatment of IS. As described previously, α-SMA can dilate and contract blood vessels, participate in neurovascular coupling, and regulate CBF (Cheng et al., 2018). Most of the pericytes that differentiated by TGFβ expressed functional α-SMA and higher levels of permeability factors such as VEGF, MMP-2, and MMP-9, indicating that TGFβ-induced differentiation of pericytes had an angiogenic effect (Thanabalasundaram et al., 2011). Nuclear factor of activated T-cells cytoplasmic 3 (NFATc3), a calcium-dependent transcriptional factor, also plays a critical role in regulating vascular cell contractility and CBF (Filosa et al., 2007; Serrano-Pérez et al., 2011). Glutamate released by neurons can activate metabotropic glutamate receptors on astrocytes, thereby increasing the expression of activated NFATc3 in pericytes, which is dependent on the activation of Ca^2+^/calmodulin-dependent phosphatase calcineurin (Filosa et al., 2007). In a study of Filosa et al., they inhibit the activation of calcineurin by using gliotoxin l-α-aminoadipic acid, and found that the NFATc3 nuclear accumulation in pericytes is prevented. This results shows that Ca2+/calcineurin is vital between mGluR and NFATc3 (Filosa et al., 2007). Based on these results, NFATc3 can be regarded as a new therapeutic target for IS.

### Regulating the Recruitment of Pericytes

Studies have shown that pericytes play a key role in the formation and maintenance of BBB (Daneman et al., 2010; Winkler et al., 2012). Pericytes are recruited to new blood vessels during early embryogenesis, which induces the formation of a functional BBB (Daneman et al., 2010). Therefore, regulating pericyte recruitment to promote angiogenesis can be regarded as a strategy to treat IS. Neuron-glial 2 (NG2) is an important component of activated pericyte-like cells (PCs). NG2 is expressed by microvascular pericytes in newly formed blood vessels under both physiological and pathological conditions (Fukushi et al., 2004; Huang et al., 2010; Yang et al., 2013). Research on angiogenesis in cancer has shown that NG2 plays a key role in pericyte recruitment and interaction with ECs during microvascular development, which can affect the maturation of pericytes and ECs (Chekenya et al., 2002; Ozerdem et al., 2002; Ozerdem and Stallcup, 2004; You et al., 2012). Another study demonstrated that CD146, a single-cell receptor, could mediate pericyte recruitment to cerebrovascular ECs and help in angiogenesis by functioning as a co-receptor for PDGFR-β (Chen et al., 2017). These results demonstrate that NG2 and CD124 may be therapeutic targets for IS.

### Pericyte-Like Cell Transplantation

Since neurogenesis is always accompanied by angiogenesis, factors that contribute significantly to angiogenesis such as PDGFRβ, VEGF, and Notch proteins could regulate neurogenesis, remodel the NVU, and treat BBB dysfunction (Farahani et al., 2019). A recent study demonstrated that human pluripotent stem cells (hPSCs) can differentiate into PCs. Further, PCs can express typical pericyte markers including PDGFRβ and CD146 (Sun et al., 2020). In this study, hPSC-induced PCs were transplanted into a transient middle cerebral artery occlusion mouse model, and the PCs promoted neurogenesis by rebuilding BBB integrity and preventing neuronal apoptosis. This result indicated that hPSC-induced PCs may be an ideal cell source for IS treatment.

### Epigenetic Regulation of Pericytes

Epigenetic mechanisms such DNA methylation, histone modification, post-transcriptional regulation of microRNAs (miRNAs), and non-coding RNA interference, are widely involved in pathophysiological processes after the occurrence of IS such as apoptosis, inflammation, BBB regulation, and neuro-angiogenesis. The role of miRNAs in the NVU after IS is being researched extensively. miRNAs can regulate endothelial function and angiogenesis, but their significance in pericyte biology has not been determined (Caporali et al., 2017). Wan et al. (2018) found that the level of miR-149-5p in pericytes decreases during IS. They also showed that *in vivo* and *in vitro*, miR-149-5p could significantly upregulate sphingosine-1-phosphate receptor 2 (S1PR2) expression in pericytes after IS. S1PR2 increased the expression of N-cadherin, which decreased pericyte migration and BBB permeability. Another study indicated that miR-532-5p was upregulated early on in murine muscular pericytes after experimentally-induced ischemia. The overexpression of miR-532-5p increased pericyte coverage by inhibiting BACH1 and promoting the expression of Ang-1 and vascular maturation *in vivo* (Slater et al., 2018). Additionally, Let-7 miRNA is involved in pericyte differentiation in response to hypoxia (Esen et al., 2016). Under conditions that mimic hyperglycemia and ischemia, pericytes can reduce capillary coverage and increase capillary permeability by absorbing endothelial cell-derived miR-503 (Caporali et al., 2015). Therefore, blocking miR-503 can increase the coverage of capillary vessels and reduce BBB permeability. In contrast, stroke can significantly alter the expression profile of long non-coding RNAs (lncRNAs) in the brain (Yan et al., 2015). However, research on the relationship between lncRNAs and pericytes is currently in the exploratory stage. Recent studies have found that hypoxia can upregulate the expression of hypoxia-induced endoplasmic reticulum stress regulating lncRNA in pericytes and regulate the differentiation, proliferation, and recruitment of pericytes to ECs (Bischoff et al., 2017). Since research on miRNAs and lncRNAs in pericytes is still in its infancy, it is necessary to carry out research in this field to explore their use as a target for IS treatment.

## Conclusion

With increased attention on NVU in recent years, the focus of IS research has gradually shifted from neuroprotection to the interaction of various components of NVU. Pericytes are not just the supporter cells of ECs, but a key component of the NVU; hence, their role in IS is being extensively studied. Pericytes are involved in the destruction and repair of the BBB after IS, immune inflammatory response, CBF regulation, angiogenesis, and epigenetic mechanisms. Pericytes perform a two-way regulatory role. The inflammation and detachment of pericytes after IS can aggravate BBB damage. Simultaneously, pericytes can play an effective role in nerve recovery by stabilizing the BBB, releasing neurotrophic factors, and promoting angiogenesis and neurogenesis. Therefore, pericytes are promising therapeutic targets for IS.

Current possible therapeutic strategies targeting pericytes include promoting the proliferation and maturation of pericytes by moderating MMP, RSG5, the Ang/Tie family, Notch1 and Notch 3 pathways, and GPR124; reducing the death and survival of pericytes by regulating HIF-1, σ-1R, VEGF, and PDGF/PDGFR; regulating the recruitment of pericytes; controlling the regulation of CBF by targeting α-SMA and NFATc3; and PC transplantation. Besides, oxidative stress plays an important role in ischemia/reperfusion after IS, and ROS may lead to pericyte loss, BBB disruption, and subsequently, intracerebral microvascular dysfunction. Against this process of oxidative stress, free radical scavengers can be considered as one of the potential therapeutic strategies for IS. Several medications can preserve pericyte function after IS. For example, edaravone, functions as a free radical scavenger, inhibits the production of MMP-9, restores the number of PDGFR-B-positive pericytes, protects the integrity of the NVU, and reduces damage after IS in rat models. Another drug, the antiplatelet drug cilostazol can promote the proliferation of pericytes, inhibit the production of MMP-9, and increase the expression of VEGFR2 in ECs, thereby maintaining BBB integrity and promoting angiogenesis after IS. Moreover, recent studies have emphasized the success of transplantation of hPSCs into ischemic areas. Similarly, transplantation of pericytes or pericyte progenitor cells can promote tissue survival or NVU reconstruction. Therefore, future research is necessary to evaluate the therapeutic efficacy of pericyte transplantation, which will greatly promote research on the therapeutic effects of pericytes in IS.

However, some drawbacks persist in targeting pericytes for IS therapy, such as understanding the function and mechanism of pericytes in the physiological and pathological conditions of the brain and the interaction between pericytes and other cells in the NVU. The pathways that regulate functions and interactions of the components of NVU are not yet completely understood. Hence, there is still a lack of clinical research and evidence for using pericytes therapeutically. Therefore, further clinical studies are needed to explore the role of pericytes in IS. Addressing these drawbacks will help researchers formulate targeted and effective treatment strategies.

## Author Contributions

S-YZ searched the literature and drafted the manuscript. Z-NG, D-HZ, YQ, and HJ critically revised the manuscript. All authors have made contributions to the work and approved it for publication.

## Conflict of Interest

The authors declare that the research was conducted in the absence of any commercial or financial relationships that could be construed as a potential conflict of interest.

## Publisher’s Note

All claims expressed in this article are solely those of the authors and do not necessarily represent those of their affiliated organizations, or those of the publisher, the editors and the reviewers. Any product that may be evaluated in this article, or claim that may be made by its manufacturer, is not guaranteed or endorsed by the publisher.
